# Photobiomodulation Therapy on Orthodontic Movement: Analysis of Preliminary Studies with a New Protocol

**DOI:** 10.3390/ijerph17103547

**Published:** 2020-05-19

**Authors:** Alessandra Impellizzeri, Martina Horodynski, Riccardo Fusco, Gaspare Palaia, Antonella Polimeni, Umberto Romeo, Ersilia Barbato, Gabriella Galluccio

**Affiliations:** Department of Oral and Maxillofacial Sciences, “Sapienza” University of Rome, 00161 Rome, Italy; martinahorodynski@gmail.com (M.H.); riccardo.fusco@gmail.com (R.F.); gaspare.palaia@uniroma1.it (G.P.); antonella.polimeni@uniroma1.it (A.P.); umberto.romeo@uniroma1.it (U.R.); ersilia.barbato@uniroma1.it (E.B.); gabriella.galluccio@uniroma1.it (G.G.)

**Keywords:** PBMT, photobiomodulation therapy, accelerated orthodontic movement, teeth extraction, premolar extraction, ectopic canines

## Abstract

This study aimed to investigate the effectiveness of photobiomodulation therapy (PBMT) on the acceleration of orthodontic movements, deriving from its biostimulating and regenerative capacity on soft tissues, consequent to the increase in differentiation, proliferation, and activity of cells that are involved with alveolar bone remodeling. The present randomized controlled trial was conducted on six patients who required extractive orthodontic therapy because their ectopic canines had erupted. A total of eight canines were analyzed, four of which received laser irradiation (i.e., experimental group). Two weeks after the extractions, all canines of the experimental and placebo groups were distalized simultaneously and symmetrically with the laceback retraction technique. The PBMT protocol consisted of four cycles of laser applications, one each on days 0, 3, 7, and 14 of the study, with session treatment durations of 2–4 min. The results of the descriptive analysis on the distal displacement speed of the canines after 1 month of follow-up indicate an average displacement of 1.35 mm for the non-irradiated group and 1.98 mm for the irradiated group. Through inferential analysis, a statistically significant difference (*p* < 0.05) was found between the average speed of the irradiated canines and the control canines. The low energy density laser used in this study, with the parameters set, was found to be a tool capable of statistically significantly accelerating the distal displacement of canines.

## 1. Introduction

Orthodontic treatment requires an average of 18 to 24 months of therapy. Several authors agree that these long treatment times, together with dental pain, are the main concerns for patients who undergo the treatment and are among the most important reasons causing them not to undertake it or to interrupt it [1,2].

Additionally, long treatment times are detrimental for clinicians, as they reduce the cooperation of patients [3] and increase the risk of gingivitis, caries, and external root resorption [4,5,6].

In light of these considerations, this study arose from the necessity to find a method that reduces the duration of orthodontic treatment but at the same time, was not invasive, was free of side effects, was simple to use, and that can become part of the orthodontist’s therapeutic tools.

The orthodontic research has moved its attention to low-level laser therapy (LLLT), also known in recent years as photobiomodulation therapy (PBMT) [7,8,9]. Therefore, this pilot study investigated PBMT as a potential method that could meet these needs.

According to our experience, the reduction of treatment time in orthodontics requires an acceleration of the dental movements. This can be achieved through methods that speed up the biological phenomena behind movements or by improving the mechanics and the orthodontic techniques.

Over the past few decades, technology and science have made self-ligating or “low-friction” equipment available to orthodontists, which is more efficient and capable of reducing treatment times. However, these modern biomechanical devices may have reached their maximum efficiency limit [1,2].

For this reason, orthodontic research is increasingly focusing on the development of new methods that can accelerate dental movements, together with the continuous progress in understanding the biological and molecular aspects of orthodontic treatment. 

To date, surgical, pharmacological, and physical approaches are the methods proposed the most often in the literature for increasing dental movements, and although they have shown positive results, some disadvantages prevent them from being applied in routine clinical practice. Pharmacological therapy with cytokine, Parathormone PTH, vitamin D, and RANKL/RANK/OPG show promising results [10,11,12,13], while on the other hand, relaxin reveals disadvantages, not by accelerating dental movement [14], but by increasing tooth mobility. In contrast, surgical approaches have the most predictable outcomes but with limited application due to their aggressiveness [15]. In particular, the piezocision technique is considered one of the best surgical approaches because it produces a good periodontal tissue response and an excellent aesthetic outcome [16]. The disadvantage of these surgical techniques, in addition to the invasiveness, is that the acceleration only occurs in the first 3 to 4 months and it declines with time to the same level as the controls [17,18]. PBMT has shown positive outcomes, but further investigations should be done to find the best energy and duration to achieve the highest success rate [19,20,21].

Orthodontic tooth movement occurs in the presence of mechanical stimuli involving remodeling of the alveolar bone and periodontal ligament (PDL). Bone remodeling is a process that involves both bone resorption on the pressure site and bone formation on the tension site [22]. Orthodontic tooth movement can be controlled via the size of the applied force and the biological responses from the PDL [23]. The force applied on the teeth will cause changes in the microenvironment around the PDL due to alterations in the blood flow, which leads to the secretion of different inflammatory mediators, such as cytokines, growth factors, neurotransmitters, colony-stimulating factors, and arachidonic acid metabolites. As a result of these secretions, remodeling of the bone occurs [24].

Photobiomodulation therapy (PBMT) uses a low-intensity laser such that its photon energy reaches the cell nucleus and increases the synthesis of deoxyribonucleic acid (DNA) and ribonucleic acid (RNA) and the consequent protein synthesis [25]. Furthermore, it increases the cellular mitotic activity [26]. Defined as the application of energy directly from noncoherent (light-emitting diode) or coherent (lasers) light, with wavelengths varying between 405 and 1100 nm, PBMT can produce photochemical effects by modulating cells [27,28]. Based on its stimulating and modulating properties on cellular mechanisms, the term PBMT is preferred to indicate the application of low-level laser therapy [29].

PBMT does not have a photo-thermal or photo-ablative action on tissues, in contrast to surgical lasers, but as mentioned earlier, instead has a photo-biostimulating action on cellular metabolic processes, increasing the activity of target cells [30,31], and the result of the interaction between cellular chromophores and laser radiation determines the cellular and tissue responses that translates, from a therapeutic point of view, not only into biostimulation but also into analgesic and anti-inflammatory abilities [25]. The biostimulant and analgesic effects have attracted attention in the orthognathodontic field and have induced researchers in the sector to experiment with the method for various therapeutic purposes, e.g., the acceleration of dental movements [32,33,34], the reduction of orthodontic pain [35], the increase in bone regeneration during the rapid expansion of the palate, and the increase in the stability of the anchoring miniscrews [36]. The increase in sutural bone regeneration due to PBMT found by Saito and Shimizu [36] suggests that the stimulating action of osteogenesis could also be useful for accelerating the orthodontic movement, which is based on a bone remodeling process.

It has been found that laser light stimulates the proliferation of osteoclasts, osteoblasts, and fibroblasts, and thereby affects bone remodeling and accelerates tooth movement. In particular, mechanisms involved in the acceleration of tooth movement occur due to the production of ATP and the activation of cytochrome C, as shown in Fujita et al. [32], where low-energy laser irradiation enhanced the velocity of tooth movement via RANK/RANKL and the macrophage colony-stimulating factor and its receptor expression [18].

Since this pioneering work, several studies have been carried out using PBMT to make orthodontic treatments faster and less painful. In fact, from a careful analysis of the studies carried out on both animals and patients, although contradictory results have emerged, most of them demonstrate tissue and clinical responses that encourage the application of PBMT in the orthodontic field [37,38,39,40,41].

In this context, the present study aimed at providing further clarity on the effectiveness of the low-intensity laser method in reducing orthodontic treatment durations and improving the statistical support for research to evaluate the effect of PBMT on dental movement.

## 2. Materials and Methods

### 2.1. Pilot Study Design

The present randomized controlled trial (RCT) with a single-blind “split-mouth” design was carried out at the Orthodontics Unit of the Department of Odontostomatological & Maxillo-facial Sciences of Sapienza University of Rome. The sample was reduced as it was a pilot study; however, motivated by the promising results of this study, we aim to continue our research by increasing the sample size to obtain more significant results than those that emerged in this initial study.

The study received approval from the Ethical Committee of Sapienza University of Rome (#4389) and was registered in the international public register.

Six patients who required extractive orthodontic therapy were enrolled for the trial. Three patients (2 female and 1 male), aged between 14 and 18, completed the experimental study, for a total of eight canines analyzed.

The three subjects who completed the protocol presented indications requiring the extraction of the first upper and/or lower premolars for the resolution of serious crowding [42], a protrusion (class II) [43], and a biprotrusion [44].

Of the three patients who were excluded from the study, two required the extraction of the second premolars and the third patient refused for work reasons.

All clinical cases were subjected to the normal orthognathodontic diagnostic procedure with the collection and analysis of anamnestic, clinical, and radiographic data, as well as relative standard documentation of plaster casts, orthopantomography of the arches (OPT), latero-lateral teleradiographies of the skull (TLL), and intra-oral and extra-oral photographs.

The inclusion criteria used for patient selection were the following:

- the need to extract the first upper and/or lower premolars to obtain the space necessary for the correct alignment of the arches or the distalization of the frontal sectors to restore correct antero-posterior occlusal positions (compensate for a class II or solve a bi-protrusion);
- presence of canines, second premolars, and first permanent molars in the arch;
- good periodontal health conditions;
- good level of oral hygiene;
- absence of systemic diseases or pharmacological treatments that could interfere with the orthodontic movement, such as the intake of analgesic or anti-inflammatory drugs.

Periodontal health was assessed for each patient by performing an intraoral evaluation and a periodontal probing, which allowed us to observe the absence of bleeding on probing (BOP%) and loss of clinical attachment (CAL) using a controlled (≈0.25 N) force on the apical end of the sulcus at six sites (mesio-buccal, buccal, disto-buccal, mesio-lingual, lingual, disto-lingual) on all teeth present. The oral hygiene level was measured through an evaluation of the plaque index at the same sites of all teeth (IP%). Limitations of these clinical criteria arise from a lack of standardized periodontal probes (e.g., probe dimensions, taper), examiner variability (probe pressure, angle), patient-related factors (biotype, medications, etc.), and smoking [45]. Therefore, all the periodontal probes were carried out by the same operator with the same probe (PCP UNC 15, Hu Friedy, Frankfurt, Germany), and the smoking subjects were excluded from the considered sample.

After being adequately informed about the risks and benefits expected from the experimental protocol, the patients and the responsible legal persons consented to participation in the study by signing an informed consent form.

A total of eight canines were examined, four of which were laser treated.

In each patient, the arch(es) subjected to extractions was divided into two sides or groups according to the “split mouth” design: the experimental or laser group, which corresponded to the side of the arch that received irradiation at the level of the canines, and the placebo group, which corresponded to the hemi-arch whose canines did not receive irradiation.

The choice of the right or left side of the arches to determine which of these two was the experimental group (and the contralateral for the placebo group) was random. In particular, randomization was performed for each arch using the “randomizer.org” website program.

Extraction from the left side of the maxillary arch (quadrant II) occurred in two patients as a group to be irradiated and on the right side (quadrant I) in one patient. For the only examined mandibular arch, extraction from the left side (quadrant III) occurred.

The subjects involved were not aware of the quadrant of the arch that would receive the laser treatment (single-blind RCT) since they wore dark laser-protective glasses to prevent the identification of the placebo treatment.

For both the experimental and placebo groups, the same orthodontic protocol was used, while the PBMT protocol differed between the two sub-groups.

#### 2.1.1. Orthodontic Protocol

A “straight wire” device with a slot that had a 0.022 × 0.028 section was applied to all teeth of the analyzed arches and direct bonding was carried out using Transbond XT (3M Unitek, Monrovia, CA, USA).

The orthodontic technique did not provide for the use of anchoring devices.

The orthodontic arch used was left in place for at least 1 month before the canine retraction began so it became passive and prevented unwanted dental movements.

Two weeks after the extractions, the canines were distalized simultaneously and symmetrically on the experimental and placebo sides of the arch, with a laceback retraction technique that involved the use of “eight” ligatures in 0.010 positioned under the arch and extended between the first molars and the canines.

To avoid any variation in the amount of distalizing force between the experimental and placebo sides, the laceback activations were always performed by the same operator.

The orthodontic canine retraction treatment involved two sessions of symmetrical activations.

The first activations were performed on the first day of the experimental protocol (day 0—T0) to trigger orthodontic movement.

To keep the force magnitude necessary to retract the canine constant, the laceback was reactivated after two weeks (day 14) from the first activations.

Patients were recommended to report any ligation break or displacement immediately.

The laser application began on the same day as the first laceback activations for canine retraction.

#### 2.1.2. PBMT Protocol

**Figure 1:**
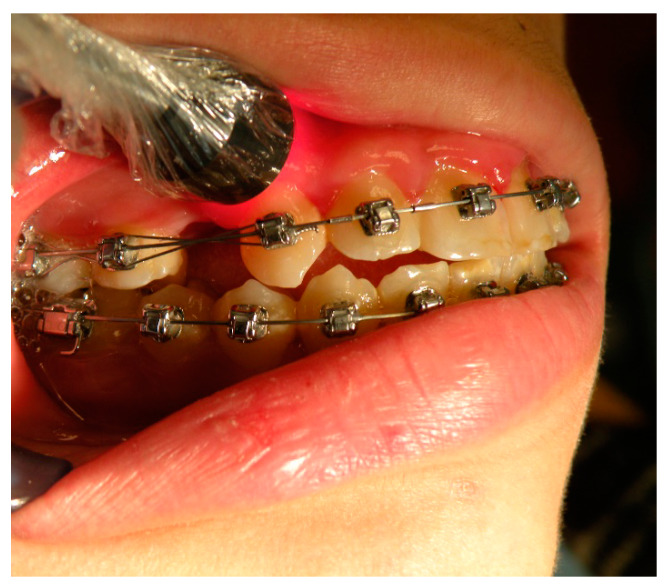
Vestibular view of the third cervical irradiation of an experimental canine.

**Figure 2:**
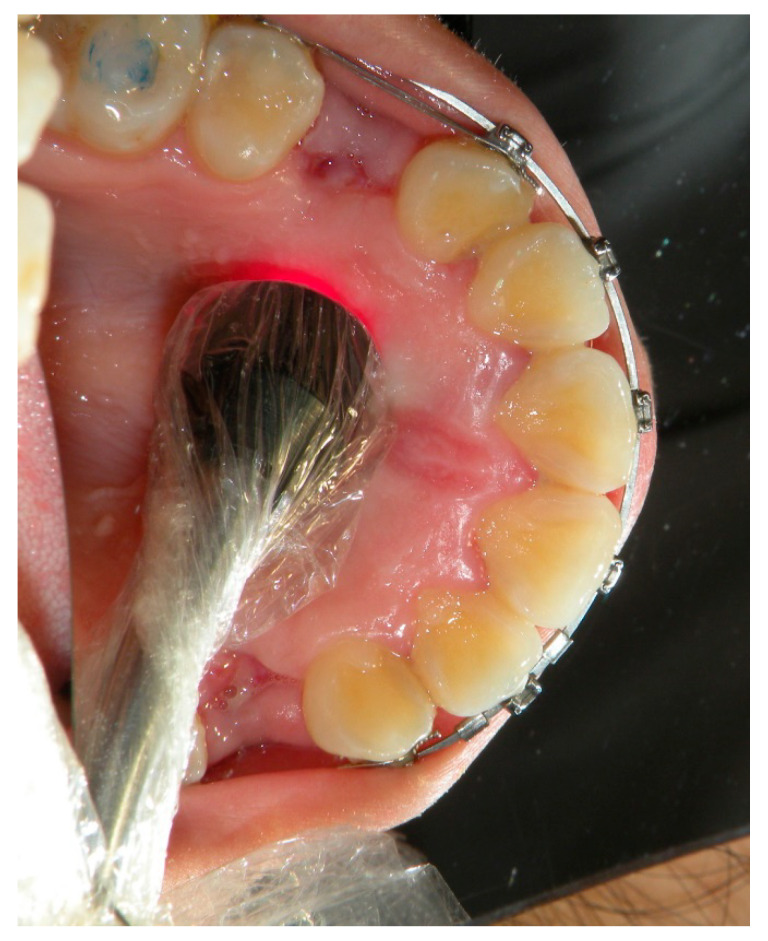
Palatal view of the third apical irradiation of the same canine in Figure 1.

The experimental canines, selected using a random method, were irradiated with an aluminum gallium arsenide (GaAlAs) double-diode laser (LUMIX 2, Fisioline, Verduno, Italy) that simultaneously emitted two wavelengths at 650 nm and 910 nm (Figure 1 and Figure 2).

The visible red source (650 nm) operated in continuous wave mode with an average power of 100 mW, while the infrared one operated in super-pulsed mode (kHz) with an average power of 500 mW and a peak power of 45 W.

The energy density (fluence) emitted by the device was set at 2 J/cm^2^.

Before starting the laser application, all precautionary measures for the patients and the operator were respected. Laser protective glasses were worn by both the operator and the patient.

The PBMT protocol involved four sessions or cycles of laser applications over a month, on days 0, 3, 7, and 14 of the study, with a session treatment duration of 2–4 min.

To avoid intra-operative bio-stimulation variations, all laser applications were performed by the same operator.

The first low-level laser application was performed immediately after the first orthodontic activation of the laceback on the first day of the experimental protocol (day 0). After this first cycle of applications, three more laser activations were performed, with one each on days 3, 7, and 14.

The areas chosen for the irradiation of the canine were the vestibular (Figure 1) and palatal (Figure 2) aspects of the periodontal ligament for a total of six points for the laser application: three palatal and three vestibular points at the apical, middle, and cervical third level of the canine root (Table 1).

All irradiations were applied with an exposure time of 10 s for each point through an 8 mm diameter fiber optic tip and a 0.5 mm^2^ “beam” area.

The total energy density (total irradiation dose) emitted in each laser session was therefore 12 J/cm^2^, and for the entire laser protocol, it was 48 J/cm^2^. This value was obtained by multiplying the energy density by the number of application points and by the number of sessions (2 J/cm^2^ × 6 points × 4 sessions = 48 J/cm^2^).

The irradiations on both the palatal and vestibular sides were carried out using the same application technique, which can be defined as “touch” because the tip of the device was in perpendicular contact with the mucous tissue but without causing pressure during the laser radiation emission.

The same procedure (points, duration of laser applications, etc.) applied on the experimental side, was also repeated in the contralateral quadrant (placebo group), but with the placement of a screen (aluminum foil) on the tip of the laser to prevent the interaction of irradiation with the tissues, and at the same time, reproduce the same conditions of the application. In this way, and by wearing dark laser-protective glasses, the possibility that the patient could identify the side of the arch subjected to irradiation was prevented. At each appointment, the Visual Analog Scale (VAS) was submitted, both to the patients of the experimental group and to the placebo group, and none of them registered pain on this scale.

### 2.2. Methods

To evaluate the speed of the orthodontic movement of the canine and the loss of anchorage, linear measurements were performed on the plaster casts of the arches.

The examined parameters were:

- the anterior–posterior position of the canines to obtain their retraction speed;
- the anterior–posterior position of the first molars to evaluate the possible loss of anchorage of the posterior sector.

The measurements of these two parameters were carried out at the beginning (T0) and at the end (T2) of the laser-orthodontic experimental protocol to evaluate the difference between the movement speeds of the irradiated canines (experimental group) and the non-irradiated canines (placebo group), as well as the possible loss of anchorage within the two groups.

At time T0, which corresponded to day 0, the first impressions were taken for the development of the initial plaster casts, immediately before the orthodontic activation with the laceback.

At time T2, which coincided with the last day of the experimental follow-up, the second impressions were made for the development of the final casts.

**Figure 3:**
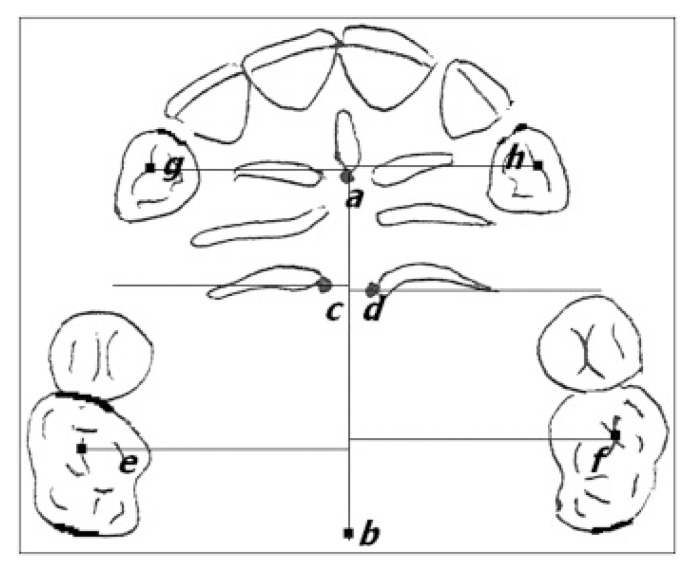
Landmark points.

**Figure 4:**
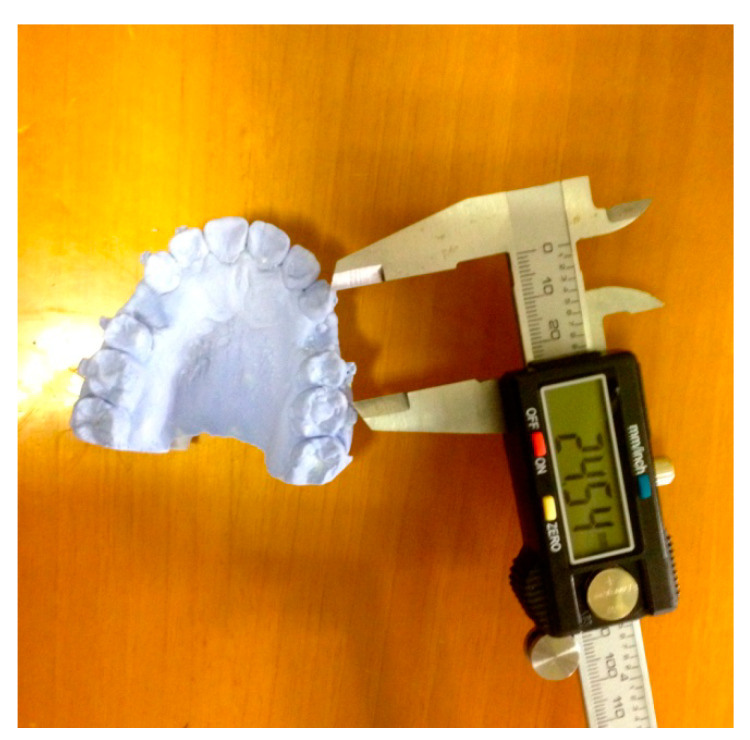
Digital caliper used for the dental movement measurement.

To measure the position of the canines and molars, the following landmarks and anatomical structures on the plaster casts were considered (Figure 3):

- cusp of the canine;
- mesio-vestibular cusp of the first molar;
- median palatine raphe;
- most medial point of the third palatine wrinkle (points C and D);
- central fossa of the first molars (points E and F);

The distal movement of the canine was measured using a digital caliper to find the linear distance in millimeters from the cusp of the canine to the mesio-vestibular cusp of the first molar (Figure 4).

The greater the dental movement, the lower the linear distance between these two anatomical points.

To evaluate the possible loss of anchorage, measurements of the linear distance in millimeters between the orthogonal projections on the palatal raphe, between points D and F in the left maxillary arch, and between points C and E in the right one were made with a millimeter “template” superimposed on the plaster cast.

The presence of anchorage loss presupposed a mesial movement of the molars and therefore a smaller distance between the anatomical points projected on the raphe, while its absence represented the invariability of this distance.

To measure the method error when using the plaster casts, Dahlberg’s formula was adopted.

## 3. Results

The results of the descriptive analysis of the two study groups, experimental and control, regarding the distal displacement speed of the canines and the loss of anchorage of the upper molars are shown below in Table 2 and Table 3, respectively.

From the results of the descriptive and inferential analysis of the loss of anchorage, no statistically significant difference between the distances of the experimental and control molars at T0 and T2 (*p* > 0.05) was observed, nor a significant difference in the mesial displacement of the first molars (loss of anchorage).

Since there was no loss of anchorage, the data relating to the movement of the canines were not altered by the study parameter. The results of the descriptive analysis on the distal displacement speed of the canines at the 1-month follow-up indicated an average displacement of 1.35 mm for the non-irradiated group and 1.98 mm for the irradiated group.

Table 3 shows the inferential analysis with Student’s *t*-test of the anchorage loss within the control group and the experimental group.

Table 2 shows the inferential analysis with Student’s *t*-test between the average displacement speed of the canines of the experimental group and the canines of the control (significance for difference T0–T1 between the two study groups).

Through the inferential analysis, a statistically significant difference (*p* < 0.05) was found between the average speed of the irradiated canines and the control canines (Table 2), with a greater increase in the movement of the canines that were subjected to irradiation.

The amount of differential speed between the two groups was 0.63 mm, which corresponded to an acceleration rate of the experimental canines of 32% compared to the control (biostimulating factor).

An analysis of the literature was carried out to evaluate the laser parameters to be used in our experimental protocol and the frequency of application of the irradiations [33,34,35,36,38,39,40,41]. All the parameters are shown in Table 4.

## 4. Discussion

This study aimed to investigate the effectiveness of PBMT on the acceleration of orthodontic movements, deriving from its biostimulating capacity, consequent to the light stimulation of osteoclasts, osteoblasts, and fibroblasts, which increase in differentiation, proliferation and activity, and begin alveolar bone remodeling. A low-level laser can be a very useful device for the acceleration of tooth movement since it increases bone remodeling without side effects to the periodontium.

The measurement of the speed of orthodontic movement of the canine was performed, as in the studies of Cruz et al. [33], Youssef et al. [34], Meta et al. [35], and Impellizzeri et al. [37], with a digital caliper measuring the linear distance between the cusp of the canine and the mesio-vestibular cusp of the first molar on the plaster casts. The possible loss of anchoring was also considered because the reduction of the post-extraction space could be determined not only by distalization of the canine but also by mesialization of the posterior sector, making the statistical survey of this variable important for the reliability of the results.

The laser device used in the present experimental research has standard programs for different therapeutic applications but none for orthodontic movement. The main application of this laser found in the literature is in oral pathology for the treatment of the burning mouth syndrome (BMS), which is clinically characterized by burning sensations in the tongue or other oral sites, often without clinical or laboratory findings [46].

The review of the literature shows that, despite the positive results of most studies in terms of the biostimulation of movement, divergent outcomes can be attributed to the various PBMT parametric protocols used [33,34,35,36,37,38,39,40,41].

For this reason, an effective PBMT protocol for dental movement was developed and tested based on an overall evaluation of the studies to provide greater reliability. Although the protocol has only been applied to this pilot study, we have obtained positive results, and by extending the sample in the future, we aim to validate this protocol or improve it to achieve more significant results.

In particular, the parameters to be defined in a PBMT protocol are divided into the irradiation parameters (wavelength and average emission power) and energy dose parameters (energy density, energy, treatment time, and radiation frequency) [24].

Among the energy parameters, the total energy delivered to the tooth and the radiation frequency were subjected to specific analysis. The total energy supplied was considered instead of the fluence due to the various energy densities proposed in the literature [33,34,35,36,37,38,39,40,41], which tend to confuse the operator regarding the effective parameters to be applied; furthermore, total energy supplied is a simpler and more easily standardized value.

Therefore, the defined protocol was characterized by a total energy of 12 J delivered to the canine in one session and an irradiation protocol of 4 sessions on days 0, 3, 7, and 14 for 1 month of experimental follow-up.

This frequency was decided upon knowing that all biostimulant protocols require repeated application of the laser as the biological effect, once activated, must be maintained. Based on a weighted average of the revised studies that had positive effects, a frequency of 4 sessions in 15 days was adopted. Limpanichkul et al. [38], who provided irradiation for the first three days from the application of the orthodontic retraction, did not find any biostimulation of the movement, suggesting that the time between the laser application is also important for the success of the treatment. For the choice of the total energy parameter, different values were used in the studies analyzed [33,34,35,36,37,38,39,40,41], from 2 J to 60 J, and no results emerged that suggest that a greater power favors the acceleration of the dental movement. Based on these data, an intermediate energy supply between the aforementioned values was chosen, assuming that this energy level could produce greater stimulating effects, without exceeding the critical threshold between a maximum accelerator effect and the inhibition of movement. According to some authors [30,31], the light stimuli follow the Arndt–Schulz law, (biphasic dose–response relationship), where the light stimuli do not produce stimulatory effects if emitted below a certain energy dose. Meanwhile, if applied above the recommended doses, an inhibitory effect is produced. Therefore, a value within the right range must be chosen. To allow for a homogeneous distribution of the energy supplied at the level of the canine periodontal support, a six-point irradiation was adopted (i.e., three palatal and three vestibular), and to obtain an amount of energy equal to 2 J per point, an irradiation time of 10 s for each point was set. Based on the exposure time, the power, and the spot diameter of the device, the fluence was 0.39 J/cm^2^, which is within the range of values suggested by the literature, i.e., between 0.01 and 10 J/cm^2^. The wavelengths of 650 nm and 910 nm emitted simultaneously by the device also fall within the “therapeutic window” of PBMT (600–1400 nm). However, infrared (IR) radiation acts deeper than red rays, making them more suitable for the biostimulation of periodontal tissues since there is a greater probability of stimulating periodontal cells located below the soft gingival tissues. Despite this important biological consideration, an analysis of the literature reveals that six studies report the positive effect of using IR; meanwhile, two other studies in which red radiation was adopted suggest that even red rays penetrate sufficiently to stimulate the periodontium. It was decided to use a wavelength of 650 nm, which was also used in one of the studies considered [40] since it obtained acceleration effects of orthodontic movement similar to those obtained using longer wavelengths. It is worth highlighting here that by simultaneously emitting red and infrared light, the adopted double-diode laser allowed for obtaining both superficial and deep biological effects in the periodontal tissues at the same time, with a wider possibility of tissue stimulation. Moreover, the super-pulsation of infrared radiation (30,000 Hz) associated with a high average power led to a deeper penetration than the continuous infrared sources used in previous studies (3–15 mm).

The fixed straight-wire devices that were used in the present study were left in place for at least 1 month before the canine retraction started such that they became passive and prevented unwanted dental movements during the retraction and therefore altering the results.

The duration of the experimental follow-up of 1 month was chosen based on the follow-up and data collection time intervals of the previous studies [33,34,35,36,37,38,39,40,41].

The choice to collect data at the end of each month has practical reasons that allow for the standardization of protocols and the possibility of comparing the results with more reliable data from a statistical point of view.

The comparison of the estimated absolute value of the percentage acceleration rate of the irradiated canines compared to the control shows that in the PBMT-irradiated side, the orthodontic speed of the irradiated canines was 32% greater than the control side.

These results are in good agreement with those reported by other authors [33,34,35,39,40]; these authors have demonstrated the capacity of the PBMT to speed up the distal displacement of the canines but obtained different acceleration rates. While these results do not agree with two of the studies considered [38,41], these are studies that did not observe any effect of the laser on tooth movement.

The estimated speed rate is within the percentage rates obtained in the other studies, and specifically, higher than that of Cruz et al. [33] and Gui et al. [40], but lower than the other three [34,35,39].

Concerning the energy dose in the proposed protocol, the 12 J energy delivered to the canine should have had similar or greater effects to those of Youssef [34] and Metha [35], who delivered 8 J. However, the acceleration rate was lower than expected, confirming that 8 J is perhaps the most reliable energy value.

The variability of the results could depend not only on the variability of the parameters used but also on other factors, such as the individual tissue response and the type of target tissue of the therapy [47,48,49]. In the periodontium, which has a very small thickness (i.e., few millimeters), there are different tissues that respond differently to the various wavelengths, namely the mucous membrane, periosteum, bone, periodontal ligament, and root cement. From a clinical point of view, the acceleration of orthodontic movements could result in a reduction in treatment times, with particular advantages for patients [50,51].

## 5. Conclusions

The low-energy-density laser used in this study, with the parameters set, was found to be an efficient instrument for accelerating the distal displacement of the canines in a statistically significant way, showing an acceleration rate of the experimental group of 32% greater than the placebo group after 1 month of follow-up, which is in agreement with the pertinent literature.

From an analysis of the literature, the action mechanisms of PBMT on the orthodontic movement are not entirely clear and there are many contradictory results. Therefore, more experiments are needed to differentiate the optimal energy, wavelength, and duration for usage.

Thus, more histological studies on animal models must be performed to better understand the biological effects of the device. A chemical analysis of the gingival crevicolar fluid (GCF) for the detection of chemical mediators could be a technique that can be used to better clarify the bio-molecular aspects related to the application of PBMT.

Therefore, it can be deduced after analyzing the results of our pilot study that PBMT is effective at reducing treatment times. The acceleration rate of the irradiated canines found in this study showed a reduction in the retraction times from 30 to 20 days based on simple mathematical derivations. Finally, the tool used was well accepted by patients, not invasive, short term, and painless. It can be concluded that this method could concretely become part of the therapeutic tools of the orthodontist for the execution of faster orthodontic treatments. However, a limitation of this study is the small sample, as it is a pilot study, but we set out to continue our research by increasing the sample to obtain more significant results than those that emerged in this initial study, which are promising.

In the current state of research, the execution of faster orthodontic treatments would apply to the closure of the post-extraction spaces but could also apply in other clinical cases, such as the distalization of the frontal group or the surgical-orthodontic disinclusion.

## Figures and Tables

**Table 1:** PBMT protocol performed on the vestibular and palatal sides of the canine’s root for a total of six points for the laser application. The same protocol was repeated on days 3, 7, and 14. PBMT PROTOCOL.

Step 1: Laser Application on Vestibular Side	Step 2: Laser Application on Palatal Side
Apical third (10 s)	Apical third (10 s)
Medium third (10 s)	Medium third (10 s)
Cervical third (10 s)	Cervical third (10 s)

**Table 2:** Average Distal Displacement Entity of Canines (mm) after 1 Month of Follow-Up (Difference T0 − T2).

Variables	Canine Displacement Data	
	Control Group	Experimental Group
Average at T2 (after)	1.35 ± 0.1782 mm	1.98 ± 0.3321 mm
*t*-test	3.3563	
Degrees of freedom	6	
*p* (level of significance)	0.0153	

**Table 3:** Average Position of the First Molars (mm) Before (T0) and After 1 Month of Follow-Up (T2).

Variables	Molar Displacement data	
	Control Group	Experimental Group
Average at T0 (before)	10.70 ± 1.7792 mm	10.94 ± 2.3209 mm
Average at T2 (after)	10.58 ± 1.7016 mm	10.88 ± 2.3386 mm
*t*-test	0.0821	0.0315
Degrees of freedom	4	4
*p* (level of significance)	0.9385	0.9763

**Table 4:** Analysis of the literature.

Study	Type of Laser/Rad Emission Mode	Wave Lengh (nm)	Power (mW)	Total Energy (J/tooth)	Energy Density (J/cm^2^)	Frequency of Laser Applications (For Each Month)	Acceleration Rate (Per Month in %)	Follow Up Month
**Cruz [33]** **2004**	GaAIAs diode/continuous	780	20	2	5	0, 3, 7, 14/ Month	17	2
**Sousa [39]** **2011**	GaAIAs diode/continuous	780	20	2	5	0, 3, 7/ month	64	3
**GUI [40]** **2008**	GaAIAs diode/continuous	650	20	Not specified	25	0, 7, 14, 21/ month	22	1
**Youssef [34]** **2008**	GaAIAs diode/continuous	809	100	8	8	0, 7, 14, 21/ month	49	6
**Doshi-Metha [35] 2012**	GaAIAs diode/continuous	810	0.25	9	20	0, 3, 7, 14/ month	56	3
**Limpanichkul [38] 2006**	GaAIAs diode/continuous	860	100	18.4	25	0, 1, 2, 3/ month	No acceleration	3
**Heravi [41]** **2014**	GaAIAs diode/continuous	810	200	60	21.4	0, 3, 7, 11, 15/ month	No acceleration	3
**Experimental Protocol**	GaAIAs bi- diode/continuous and superpulsed	650/910	100/500	12	0.4	0, 3, 7, 14/ month	32	1

## References

[1] Fink D.F., Smith R.J. (1992). The duration of orthodontic treatment. Am. J. Orthod. Dentofac. Orthop..

[2] Skidmore K.J., Brook K.J., Thomson W.M., Harding W.J. (2006). Factors influencing treatment time in orthodontic patients. Am. J. Orthod. Dentofac. Orthop..

[3] Impellizzeri A., Putrino A., Zangrillo C., Barbato E., Galluccio G. (2019). Efficiency of self-ligating vs conventional braces: Systematic review and meta-analysis. J. Dent. Cadmos.

[4] Segal G.R., Schiffman P.H., Tuncay O.C. (2004). Meta analysis of the treatment-related factors of external apical root resorption. Orthod. Craniofacial Res..

[5] Pandis N., Nasika M., Polychronopoulou A., Eliades T. (2008). External apical root resorption in patients treated with conventional and self- ligating brackets. Am. J. Orthod. Dentofac. Orthop..

[6] Impellizzeri A., Di Benedetto S., De Stefano A., Guercio E.M., Barbato E., Galluccio G. (2019). General health & psychological distress in children with temporomandibular disorder. Case Report. Clin. Ter.

[7] Pomini K.T., Buchaim D.V., Andreo J.C., Rosso M.P.D.O., Della Coletta B.B., German I.S., Bighetti A., Shinohara A., Junior G.M.R., Shindo J. (2019). Fibrin Sealant Derived from Human Plasma as a Scaffold for Bone Grafts Associated with Photobiomodulation Therapy. Int. J. Mol. Sci..

[8] Rosso M.P.D.O., Junior G.M.R., Buchaim D.V., German I.J.S., Pomini K.T., De Souza R.G., Pereira M., Júnior I.A.F., Bueno C.R.D.S., Gonçalves J.B.D.O. (2017). Stimulation of morphofunctional repair of the facial nerve with photobiomodulation, using the end-to-side technique or a new heterologous fibrin sealant. J. Photochem. Photobiol. B: Boil..

[9] Zein R., Selting W., Hamblin M. (2018). Review of light parameters and photobiomodulation efficacy: Dive into complexity. J. Biomed. Opt..

[10] Collins M.K., Sinclair P.M. (1988). The local use of vitamin D to increase the rate of orthodontic tooth movement. Am. J. Orthod. Dentofac. Orthop..

[11] Seifi M., Eslami B., Saffar A.S. (2003). The effect of prostaglandin E2 and calcium gluconate on orthodontic tooth movement and root resorption in rats. Eur. J. Orthod..

[12] Kale S., Kocadereli I., Atilla P., Asan E. (2004). Comparison of the effects of 1,25 dihydroxycholecalciferol and prostaglandin E2 on orthodontic tooth movement. Am. J. Orthod. Dentofac. Orthop..

[13] Soma S., Matsumoto S., Higuchi Y., Takano-Yamamoto T., Yamashita K., Kurisu K., Iwamoto M. (2000). Local and chronic application of PTH accelerates tooth movement in rats. J. Dent. Res..

[14] Liu Z.J., King G.J., Gu G.M., Shin J.Y., Stewart D.R. (2005). Does Human Relaxin Accelerate Orthodontic Tooth Movement in Rats?. Ann. N. Y. Acad. Sci..

[15] Ren A., Lv T., Kang N., Zhao B., Chen Y., Bai D. (2007). Rapid orthodontic tooth movement aided by alveolar surgery in beagles. Am. J. Orthod. Dentofac. Orthop..

[16] Liou E.J., Huang C. (1998). Rapid canine retraction through distraction of the periodontal ligament. Am. J. Orthod. Dentofac. Orthop..

[17] Wang L., Lee W., Lei D.-L., Liu Y.-P., Yamashita D.-D., Yen S. (2009). Tisssue responses in corticotomy- and osteotomy-assisted tooth movements in rats: Histology and immunostaining. Am. J. Orthod. Dentofac. Orthop..

[18] Baloul S.S., Gerstenfeld L.C., Morgan E.F., Carvalho R.S., Van-Dyke T.E., Kantarci A. (2011). Mechanism of action and morphologic changes in the alveolar bone in response to selective alveolar decortication-facilitated tooth movement. Am. J. Orthod. Dentofac. Orthop..

[19] Kau C.H.,  Kantarci A., Shaughnessy T., Vachiramon A., Santiwong P., de la Fuente A., Skrenes D., Ma D., Brawn P. (2013). Photobiomodulation accelerates orthodontic alignment in the early phase of treatment. Prog. Orthod..

[20] Kawasaki K., Shimizu N. (2000). Effects of low-energy laser irradiation on bone remodeling during experimental tooth movement in rats. Lasers Surg. Med..

[21] Nimeri G., Kau C.H., Abou-Kheir N.S., Corona R. (2013). Acceleration of tooth movement during orthodontic treatment—a frontier in Orthodontics. Prog. Orthod..

[22] Davidovitch Z. (1991). Tooth movement. Crit. Rev. Oral Biol. Med..

[23] Meikle M. (2005). The tissue, cellular, and molecular regulation of orthodontic tooth movement: 100 years after Carl Sandstedt. Eur. J. Orthod..

[24] Davidovitch Z., Nicolay O.F., Ngan P.W., Shanfeld J.L. (1988). Neurotransmitters, cytokines, and the control of alveolar bone remodeling in orthodontics. Dent. Clin. N. Am..

[25] Tim C.R., Bossini P.S., Kido H., Malavazi I., Von-Zeska-Kress M.R., Carazzolle M.F., Parizotto N.A., Rennó A.C. (2016). Effects of low level laser therapy on inflammatory and angiogenic gene expression during the process of bone healing: A microarray analysis. J. Photochem. Photobiol. B: Boil..

[26] Karu T., Pyatibrat L., Kalendo G. (1995). Irradiation with He-Ne laser increases ATP level in cells cultivated in vitro. J. Photochem. Photobiol. B: Boil..

[27] De Freitas L.F., Hamblin M. (2016). Proposed Mechanisms of Photobiomodulation or Low-Level Light Therapy. IEEE J. Sel. Top. Quantum Electron..

[28] Tani A., Chellini F., Giannelli M., Nosi D., Zecchi-Orl R., Sassoli C. (2018). Red (635 nm), Near-Infrared (808 nm) and Violet-Blue (405 nm) Photobiomodulation Potentiality on Human Osteoblasts and Mesenchymal Stromal Cells: A Morphological and Molecular In Vitro Study. Int. J. Mol. Sci..

[29] Rosso M.P.D.O., Oyadomari A., Pomini K.T., Della Coletta B.B., Shindo J., Junior R.S.F., Barraviera B., Cassaro C.V., Buchaim D.V., Teixeira D.D.B. (2020). Photobiomodulation Therapy Associated with Heterologous Fibrin Biopolymer and Bovine Bone Matrix Helps to Reconstruct Long Bones. Biomolecules.

[30] Schindl A., Schindl M., Pernerstorfer-Schön H., Schindl L. (2000). Low-intensity laser therapy: A review. J. Investig. Med..

[31] Karu T.I. (1989). Photobiology of Low-Power Laser Therapy.

[32] Fujita S., Yamaguchi M., Utsunomiya T., Yamamoto H., Kasai K. (2008). Low-energy laser stimulates tooth movement velocity via expression of RANK and RANKL. Orthod. Craniofacial Res..

[33] Cruz D.R., Kohara E.K., Ribeiro M., Wetter N. (2004). Effects of low-intensity laser therapy on the orthodontic movement velocity of human teeth: A preliminary study. Lasers Surg. Med..

[34] Youssef M., Ashkar S., Hamade E., Gutknecht N., Lampert F., Mir M. (2007). The effect of low-level laser therapy during orthodontic movement: A preliminary study. Lasers Med. Sci..

[35] Doshi-Mehta G., Bhad-Patil W.A. (2012). Efficacy of low-intensity laser therapy in reducing treatment time and orthodontic pain: A clinical investigation. Am. J. Orthod. Dentofac. Orthop..

[36] Saito S., Shimizu N. (1997). Stimulatory effects of low-power laser irradiation on bone regeneration in midpalatal suture during expansion in the rat. Am. J. Orthod. Dentofac. Orthop..

[37] Impellizzeri A., Horodynski M., Serritella E., Romeo U., Barbato E., Galluccio G. (2020). Three dimensional evaluation of dental movement in orthodontics. Dent. Cadmos.

[38] Limpanichkul W., Godfrey K., Srisuk N., Rattanayatikul C. (2006). Effects of low-level laser therapy on the rate of orthodontic tooth movement. Orthod. Craniofacial Res..

[39] Sousa M.V.S., Scanavini M.A., Sannomya E.K., Velasco L.G., Angelieri F. (2011). Influence of Low-Level Laser on the Speed of Orthodontic Movement. Photomed. Laser Surg..

[40] Gui L., Qu H. (2008). Clinical application of low energy laser in acceleration of orthodontic tooth movement. J. Dalian Med. Univ..

[41] Heravi F., Moradi A., Ahrari F. (2014). The effect of low level laser therapy on the rate of tooth movement and pain perception during canine retraction. Oral Heal. Dent. Manag..

[42] Filho H.L., Maia L.H., Lau T.C.L., De Souza M.M.G., Maia L.C. (2014). Early vs late orthodontic treatment of tooth crowding by first premolar extraction: A systematic review. Angle Orthod..

[43] De Araújo T.M., Caldas L.D. (2019). Tooth extractions in Orthodontics: First or second premolars?. Dent. Press J. Orthod..

[44] Bills D.A., Handelman C.S., A BeGole E. (2005). Bimaxillary dentoalveolar protrusion: Traits and orthodontic correction. Angle Orthod..

[45] Chapple I.L.C., Mealey B.L., Van Dyke T.E., Bartold P.M., Dommisch H., Eickholz P., Geisinger M.L., Genco R.J., Glogauer M., Goldstein M. (2018). Periodontal health and gingival diseases and conditions on an intact and a reduced periodontium: Consensus report of workgroup 1 of the 2017 World Workshop on the Classification of Periodontal and Peri-Implant Diseases and Conditions. J. Clin. Periodontol..

[46] Romeo U., Del Vecchio A., Capocci M., Maggiore C., Ripari M. (2010). The low level laser therapy in the management of neurological burning mouth syndrome. A pilot study. Ann. Stomatol. (Roma).

[47] Palaia G., Del Vecchio A., Impellizzeri A., Tenore G., Visca P., Libotte F., Russo C., Romeo U. (2014). Histological in vitro evaluation of peri-incisional thermal effect created by new-generation CO2 super-pulsed laser. Sci. World J..

[48] Impellizzeri A., Palaia G., Horodynski M., Pergolini D., Vernucci R.A., Romeo U., Galluccio G. (2020). CO2 laser for surgical exposure of impacted palatally canines. Esposizione chirurgica mediante laser CO2 di canini inclusi palatali. Dent. Cadmos.

[49] Palaia G., Impellizzeri A., Tenore G., Caporali F., Visca P., Del Vecchio A., Galluccio G., Polimeni A., Romeo U. (2019). Ex vivo histological analysis of the thermal effects created by a 445-nm diode laser in oral soft tissue biopsy. Clin. Oral Investig..

[50] Putrino A., Impellizzeri A., Pavese L., Barbato E., Galluccio G. (2019). Orthodontic treatment and third molars development: Longitudinal study on radiographs. J. Dent. Cadmos.

[51] Impellizzeri A., Midulla G., Romeo U., La Monaca C., Barbato E., Galluccio G. (2018). Delayed Eruption of Permanent Dentition and Maxillary Contraction in Patients with Cleidocranial Dysplasia: Review and Report of a Family. Int. J. Dent..

